# A nomogram to predict early spontaneous abortion in women with polycystic ovary syndrome: a retrospective cohort study

**DOI:** 10.1186/s12884-026-09641-w

**Published:** 2026-07-10

**Authors:** Qian Han, Mengyuan Li, Guanshan Li, Mingwei Xin, Xiaodan Yin, Junqin He

**Affiliations:** 1 Department of Traditional Chinese Medicine, Beijing Obstetrics and Gynecology Hospital, Capital Medical University, Beijing Maternal and Child Health Care Hospital, No. 251 Yaojiayuan Road, Chaoyang District, Beijing, 100026 China; 2 Capital Medical University, No.10 Xitoutiao, You’anmenwai, Fengtai District, Beijing, 100069 China

**Keywords:** Polycystic ovary syndrome, Early spontaneous abortion, Nomogram, Prediction model

## Abstract

**Background:**

Polycystic ovary syndrome (PCOS) is associated with a significantly high risk of spontaneous abortion, yet there is a scarcity of clinical prediction models for early pregnancy loss in this patient population.

**Objective:**

To identify risk factors for early spontaneous abortion in women with PCOS and to develop and validate a predictive model for assessing this risk.

**Methods:**

Researchers collected clinical data including demographic statistics, medical, menstrual, obstetric, and biochemical data. Univariate logistic regression was initially used to identify potential factors associated with early spontaneous abortion. Least absolute shrinkage and selection operator (LASSO) regression was applied for variable selection and dimension reduction. Multivariate logistic regression was then used to determine independent predictors, based which a nomogram was constructed. The model’s discriminative ability, clinical utility, and calibration were assessed using the receiver operating characteristic (ROC) curve, decision curve analysis (DCA), and calibration plots.

**Results:**

This retrospective study included 619 PCOS patients from a maternity and gynecological hospital in China. Univariate analysis revealed that elevated testosterone, higher HOMA-IR, increased D-dimer, decreased serum iron, platelet (PLT) count ≥ 300 × 10⁹/L, and homocysteine (HCY) > 7 µmol/L were significantly associated with an increased risk of spontaneous abortion, while early pregnancy supportive treatment reduced the risk. LASSO regression identified six key predictors: testosterone, D-dimer, serum iron, early pregnancy support, PLT count, and HCY. Multivariate analysis confirmed that elevated testosterone, increased D-dimer, PLT count ≥ 300 × 10⁹/L, and HCY > 7 µmol/L were independent risk factors, while higher serum iron was a protective factor. The nomogram developed from these factors showed high predictive accuracy, with an area under the curve (AUC) of 0.92 (95% CI: 0.90–0.95) in the training set and 0.86 (95% CI: 0.80–0.93) in the vaildation set. Decision curve and calibration analyses further demonstrated favorable clinical applicability and model fit.

**Conclusion:**

The constructed nomogram provides an accurate and clinically useful tool for predicting the risk of early spontaneous abortion in patients with PCOS, facilitating personalized treatment and early intervention strategies.

**Supplementary Information:**

The online version contains supplementary material available at https://doi.org/10.1186/s12884-026-09641-w.

## Introduction

Polycystic ovary syndrome (PCOS) is a complex endocrine and metabolic disorder characterized by heterogeneous pathogenesis involving interactions among genetic, environmental, and lifestyle factors [1]. It affects approximately 6%–20% of women of reproductive age worldwide, significantly compromising their reproductive health, metabolic profile, and quality of life [1]. The clinical manifestations of PCOS are diverse, including menstrual irregularities, hyperandrogenism, and polycystic ovarian morphology, and it is often accompanied by obesity, insulin resistance, dyslipidemia, and coagulation abnormalities [2]. PCOS is not only a major cause of infertility but also strongly associated with various pregnancy complications, such as gestational diabetes and hypertensive disorders. Moreover, it contributes to adverse pregnancy outcomes including miscarriage and preterm birth, posing serious threats to both fertility and long-term health [3].

Early spontaneous abortion, defined as the loss of pregnancy before 12 weeks of gestation, has a reported incidence ranging from 4.93% to 6.43% [4, 5]. PCOS is among the most significant endocrine disorders contributing to spontaneous abortion, with affected women having a 1.617-fold higher risk of early pregnancy loss compared to those without PCOS [6]. Some studies suggest that up to 58% of patients with recurrent spontaneous abortion may have underlying PCOS [1]. The disorder’s characteristic endocrine and metabolic abnormalities—such as hormonal imbalances, hyperandrogenism, insulin resistance, and hyperinsulinemia—contribute collectively to pregnancy loss through multiple pathways [7, 8]. Notably, many women with PCOS exhibit coagulopathies characterized by a hypercoagulable state, which can promote the formation of microthrombi in placental vessels, impair placental perfusion and oxygen supply, and thereby compromise embryonic development, increasing the risk of miscarriage [9]. Beyond its physical implications, spontaneous abortion also inflicts profound psychological distress and affects family well-being [10]. Preconception interventions have been shown to reduce the incidence of abortion in high-risk groups and improve overall pregnancy outcomes [11]. Therefore, identifying risk factors for miscarriage in patients with PCOS is essential for enhancing fertility management and prenatal care in this population.

The risk factors for spontaneous abortion in PCOS are multifactorial and complex, with studies reporting close associations with PCOS phenotypes, body mass index (BMI), hyperandrogenism, anti-Müllerian hormone (AMH), metabolic disturbances, hypercoagulability, hyperhomocysteinemia, and cervical insufficiency [6, 12, 13]. Although predictive models for live birth rates and spontaneous abortion in PCOS have been developed, most studies exploring risk factors have focused primarily on reproductive hormones and metabolic parameters, often examining only one or a few variables [14]. This approach falls short of comprehensively elucidating the complex mechanisms underlying spontaneous abortion in PCOS, thereby limiting the accurate identification of high-risk populations and effective intervention strategies. To date, only one review focusing on PCOS and recurrent spontaneous abortion has provided a detailed discussion of factors such as hyperandrogenism, polycystic ovarian morphology, thrombophilia, genetic polymorphisms, insulin resistance, and AMH as potential risk contributors [12].

Given this research background, the present study aims to identify independent risk factors for spontaneous abortion in women with PCOS using logistic regression analysis, and to develop and validate a predictive model for early pregnancy loss in this population. By comprehensively evaluating clinical characteristics, endocrine and metabolic parameters, coagulation function, and other factors influencing pregnancy outcomes, this study seeks to provide a scientific basis for the early identification of high-risk individuals and the implementation of personalized interventions for preventing early spontaneous abortion in PCOS.

## Materials and methods

### Study design

This retrospective study was conducted at Beijing Obstetrics and Gynecology Hospital, Capital Medical University. The study protocol was approved by the hospital’s Ethics Committee (Approval No. 2025-KY-047-01) prior to its initiation.

### Study participants

Patients diagnosed with PCOS at Beijing Obstetrics and Gynecology Hospital between January 2019 and May 2025 were included. PCOS was diagnosed according to the 2003 Rotterdam criteria [15]. Eligible participants were women aged 16 to 45 years with a history of pregnancy, and no restrictions were applied regarding body mass index (BMI). However, cases were excluded from the analysis if the miscarriage was attributable to embryonic chromosomal abnormalities or iatrogenic factors, or if the pregnancy termination occurred after 12 weeks of gestation. Additionally, patients with ectopic pregnancy (EP), gestational trophoblastic disease (GTD), or abortion resulting from trauma or iatrogenic causes were excluded. Those with organic gynecological conditions such as submucous uterine fibroids, endometriosis, endometrial polyps, or uterine anomalies were also omitted. Patients suffering from severe systemic diseases affecting the heart, liver, kidneys, brain, malignancy, or hematological disorders were not eligible. Cases with extensively missing clinical data were also removed.

### Data collection

Clinical data were retrieved from the electronic medical records within the Hospital Information System (HIS) of Beijing Obstetrics and Gynecology Hospital. Clinical data collected from the patients included demographic characteristics, medical histories (specifically of PCOS, other diseases, and medication), detailed gynecological and obstetric histories (encompassing menstrual, fertility, and miscarriage history), as well as biochemical parameters and pelvic ultrasound findings. Based on current research advancements, the candidate variables for statistical analysis included: menstrual history, number of previous abortions; age at delivery or abortion; pre-pregnancy BMI within three months before conception, AMH, luteinizing hormone/follicle-stimulating hormone (LH/FSH) ratio, testosterone, homeostasis model assessment of insulin resistance (HOMA-IR) index. First-trimester (within 12 weeks of gestation) parameters such as thyroid-stimulating hormone (TSH), platelet (PLT) count, homocysteine (HCY), coagulation profiles (fibrinogen [FIB], prothrombin time [PT], prealbumin [PA], thrombin time [TT], activated partial thromboplastin time [APTT], D-dimer), lipid profiles, serum iron, and the use of pregnancy-supporting medications were also collected.

The HOMA-IR index was used to evaluate insulin resistance, calculated as: HOMA-IR = Fasting Glucose (mmol/L) × Fasting Insulin (µU/mL) / 22.5 [16].

Dyslipidemia was defined as: Total cholesterol > 5.18 mmol/L, triglycerides > 1.7 mmol/L, high-density lipoprotein < 1.04 mmol/L, or low-density lipoprotein > 3.34 mmol/L. The presence of any of these abnormalities indicated disordered lipid metabolism.

Fetal protection was defined as the use of progesterone, human chorionic gonadotropin, or low-molecular-weight heparin. As this was a retrospective study, no restrictions were imposed regarding the dosage or duration of these medications.

### Data analysis

This study employed a stepwise analytical strategy to systematically identify key factors influencing early spontaneous abortion in PCOS and to construct a predictive model. After excluding samples with missing values, the dataset was randomly divided into a training set (*n* = 496) and a validation set (*n* = 126) at an 8:2 ratio using R software. A t-test confirmed the balance of baseline characteristics between the two sets, supporting the randomness of the split.

The following analytical steps were sequentially performed in the training set. First, univariate logistic regression was applied to examine the association between each independent variable and the outcome; variables with a P-value < 0.2 were retained for further analysis. To address multicollinearity and reduce overfitting, least absolute shrinkage and selection operator (LASSO) regression was introduced. This method uses L1 regularization to shrink the coefficients of less relevant variables to zero. The optimal penalty parameter was determined via 10-fold cross-validation, and variables with non-zero coefficients were retained. The variables selected by LASSO were then incorporated into a multivariate logistic regression model, with a significance level of *P* < 0.05 used to identify independent influencing factors. Finally, a predictive nomogram was constructed using the rms package. Model performance was evaluated from multiple perspectives: The receiver operating characteristic (ROC) curve was plotted and the area under the curve (AUC) was calculated to assess discriminative ability. Internal validation was performed using the bootstrap resampling method on the training set, followed by an evaluation of the model’s performance in the validation set. This comprehensive approach was used to assess the model’s predictive accuracy and clinical applicability.

## Results

**Fig. 1:**
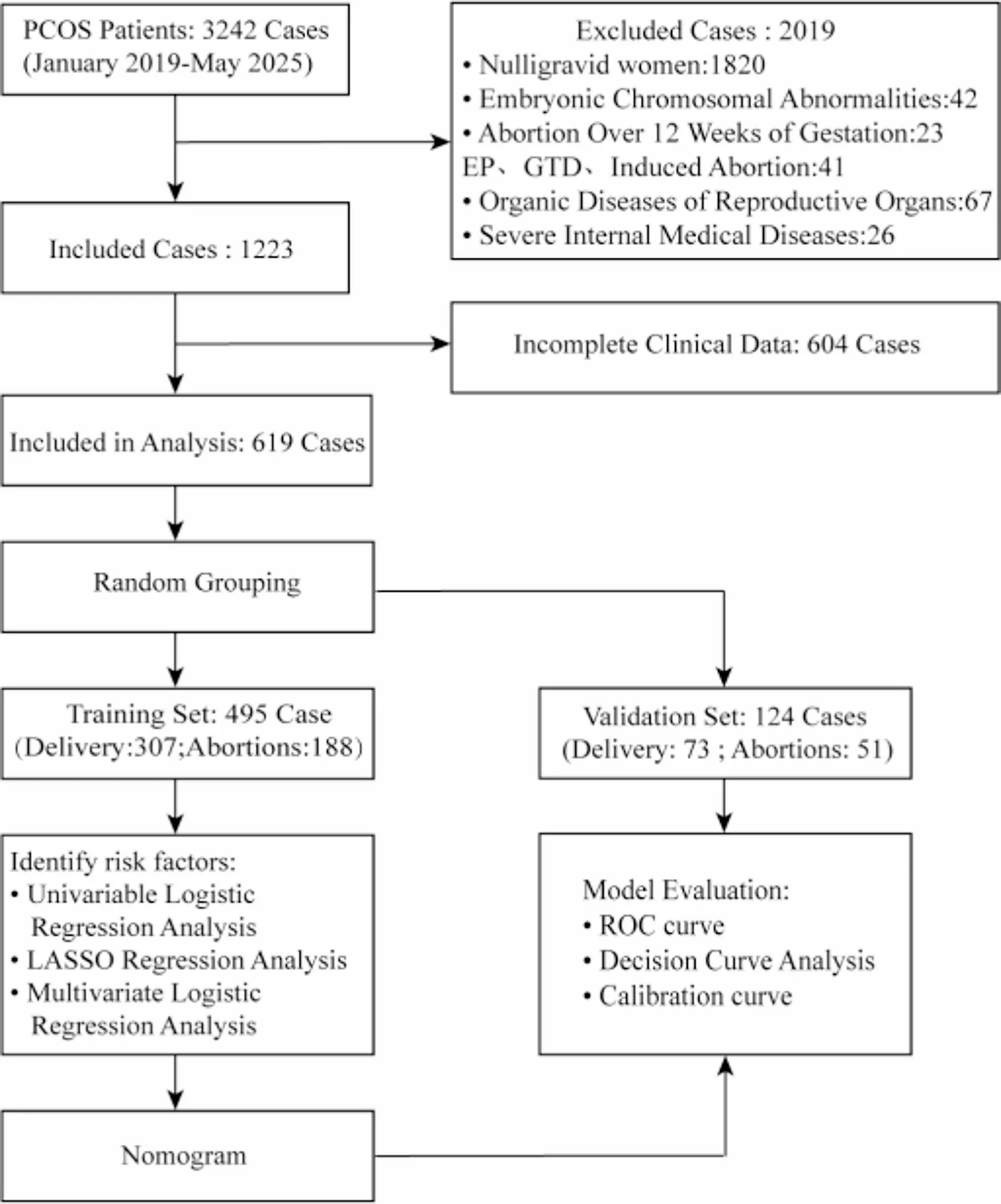
Study flowchart

From an initial 3,242 patients with PCOS, 1,223 eligible participants were identified after applying inclusion and exclusion criteria. After excluding cases with missing values, 619 patients (239 with early spontaneous abortion; 380 with healthy deliveries) were included and randomly divided into a training set (*n* = 495) and a validation set (*n* = 124) in an 8:2 ratio (Fig. 1).

### Univariate logistic regression analysis

As shown in Table 1, univariate analysis revealed seven significant associations (*P* < 0.05): elevated testosterone (OR = 51.40), HOMA-IR (OR = 1.17), D-dimer (OR = 3.58), PLT ≥ 300 × 10⁹/L (OR = 9.35), and HCY > 7 µmol/L (OR = 8.50) increased abortion risk, whereas serum iron (OR = 0.93) and fetal protection therapy (OR = 0.54) were protective. Three factors (LH/FSH ratio, FIB, and AMH > 7 ng/mL) showed borderline significance, while others, including age and BMI, were not significant. The “Number of Abortions” exhibited an unstable association requiring further validation.

**Table 1:** Univariable logistic regression analysis

Variable	Live Birth (*n* = 307)	Abortion (*n* = 188)	OR (univariable)	*P*-value
Age, Mean ± SD, years	32.0 ± 3.3	31.9 ± 3.7	0.99 (0.94–1.04)	0.652
BMI, Mean ± SD, kg/m^2^	24.6 ± 4.4	25.0 ± 4.8	1.02 (0.98–1.06)	0.327
LH/FSH, Mean ± SD	1.3 ± 0.7	1.4 ± 0.9	1.25 (1.00-1.55)	0.051
Testosterone, Mean ± SD, nmol/L	0.9 ± 0.3	1.5 ± 0.6	51.40 (24.02-109.99)	< 0.001
HOMA-IR, Mean ± SD	2.9 ± 1.8	3.6 ± 2.5	1.17 (1.06–1.28)	< 0.001
FIB, Mean ± SD, g/L	3.3 ± 0.7	3.2 ± 0.9	0.78 (0.60–1.02)	0.070
PT, Mean ± SD, S	11.4 ± 0.8	11.4 ± 0.9	1.01 (0.81–1.26)	0.932
PA, Mean ± SD, %	104.5 ± 9.4	103.5 ± 9.9	0.99 (0.97–1.01)	0.275
TT, Mean ± SD, S	16.3 ± 0.8	17.3 ± 11.4	1.17 (0.94–1.46)	0.151
APTT, Mean ± SD, S	26.9 ± 1.9	26.9 ± 1.9	1.02 (0.93–1.12)	0.669
D-Dimer, Mean ± SD, mg/L	0.3 ± 0.2	0.4 ± 0.3	3.58 (1.71–7.47)	0.001
Serum iron, Mean ± SD, umol/L	22.1 ± 6.9	18.6 ± 7.0	0.93 (0.90–0.95)	0.001
Fetal protection				
No *n*(%)	48 (15.6%)	48 (25.5%)		
Yes *n*(%)	259 (84.4%)	140 (74.5%)	0.54 (0.34–0.85)	0.007
Menstrual Irregularity				
No *n*(%)	37 (12.1%)	18 (9.6%)		
Yes *n*(%)	270 (87.9%)	170 (90.4%)	1.29 (0.71–2.35)	0.396
Number of Abortions				
0	172 (56%)	0 (0%)		
1–2	126 (41%)	156 (83%)	389215071.26 (0.00-Inf)	0.981
≥ 3	9 (2.9%)	32 (17%)	1117745845.66 (0.00-Inf)	0.980
TSH >2.5mIU/L				
No *n*(%)	226 (73.6%)	134 (71.3%)		
Yes *n*(%)	81 (26.4%)	54 (28.7%)	1.12 (0.75–1.69)	0.571
AMH >7 ng/ml				
No *n*(%)	172 (56%)	122 (64.9%)		
Yes *n*(%)	135 (44%)	66 (35.1%)	0.69 (0.47-1.00)	0.052
PLT ≥ 300 × 10^9^L				
No *n*(%)	266 (86.6%)	77 (41%)		
Yes *n*(%)	41 (13.4%)	111 (59%)	9.35 (6.03–14.51)	< 0.001
HCY >7 umol/L				
No *n*(%)	259 (84.4%)	73 (38.8%)		
Yes *n*(%)	48 (15.6%)	115 (61.2%)	8.50 (5.56–13.01)	< 0.001
Dyslipidemia				
No *n*(%)	170 (55.4%)	100 (53.2%)		
Yes *n*(%)	137 (44.6%)	88 (46.8%)	1.09 (0.76–1.57)	0.636

### LASSO regression analysis

**Fig. 2:**
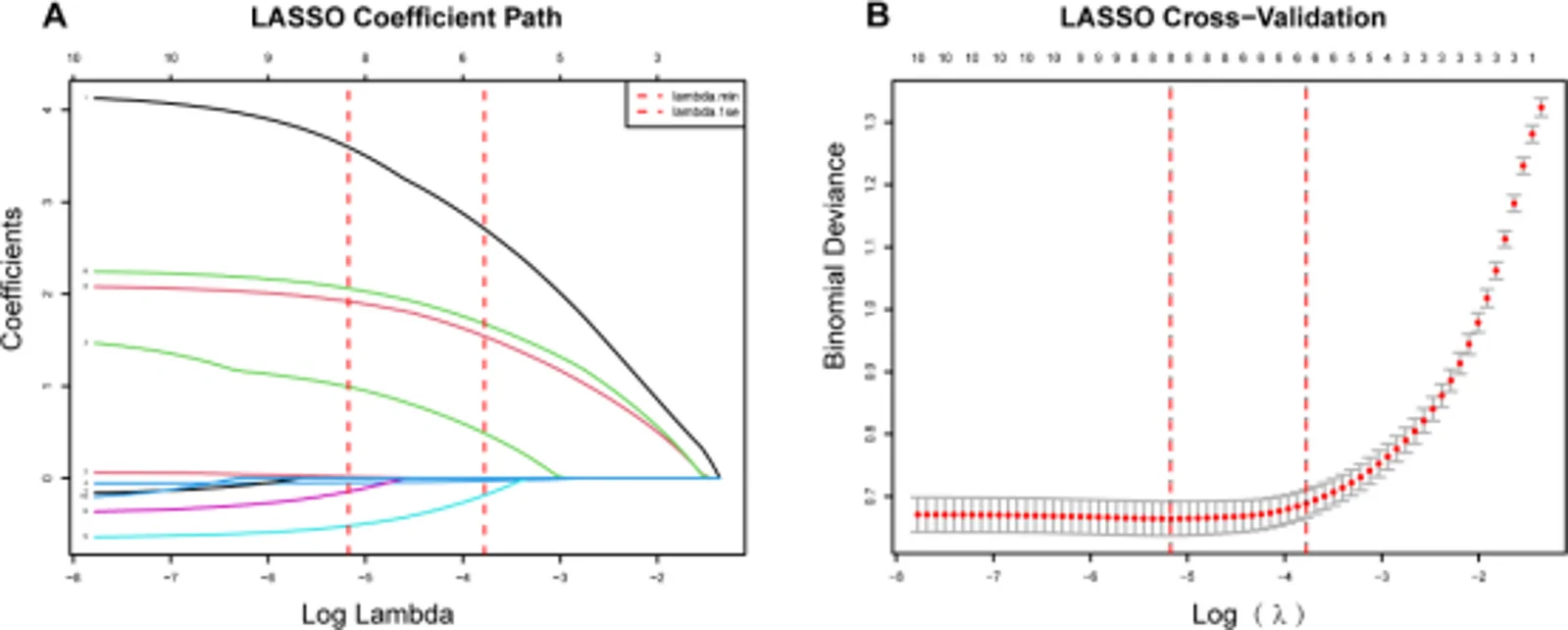
Fig. 2**A** shows the LASSO coefficient paths. As the penalty (log(λ)) increased, coefficients shrank and were selected. The vertical lines indicate the optimal λ values chosen via 10-fold cross-validation in Fig. 2**B**. The minimal binomial deviance at the selected λ confirms a stable and well-fitted model

LASSO regression identified six robust predictors of early spontaneous abortion (Table 2): testosterone, D-dimer, PLT ≥ 300 × 10⁹/L, and HCY > 7 µmol/L as positive risk factors, while serum iron and fetal protection therapy were protective. The coefficient profile (Fig. 2A) illustrates the selection process, and 10-fold cross-validation (Fig. 2B) confirms the model’s optimal fit and stability.

**Table 2:** LASSO regression analysis

Variable	Coef
(Intercept)	-4.12156281
Testosterone	2.708714526
D-Dimer	0.487871894
Serum iron	-0.028026376
Fetal protection	-0.184889736
PLT ≥ 300 × 10^9^L	1.541373901
HCY >7 umol/L	1.675800129

### Multivariate logistic regression analysis

Multivariate analysis identified five factors independently associated with early spontaneous abortion (*P* < 0.05, Table 3). Elevated testosterone (OR = 50.11), D-dimer (OR = 3.40), PLT count ≥ 300 × 10⁹/L (OR = 8.45), and HCY > 7 µmol/L (OR = 9.57) remained independent risk factors, while serum iron was protective (OR = 0.94). Fetal protection therapy showed a non-significant trend toward risk reduction (OR = 0.51, *P* = 0.057).

**Table 3:** Multivariate logistic regression analysis

Variable	Live Birth (*n* = 307)	Abortion (*n* = 188)	OR (univariable)	*P*-value
Testosterone, Mean ± SD, nmol/L	0.9 ± 0.3	1.5 ± 0.6	50.11(18.82-133.45)	< 0.001
D-Dimer, Mean ± SD, mg/L	0.3 ± 0.2	0.4 ± 0.3	3.40 (1.15–10.01)	0.027
Serum iron, Mean ± SD, umol/L	22.1 ± 6.9	18.6 ± 7.0	0.94 (0.91–0.98)	0.007
Fetal protection				
No *n*(%)	48 (15.6%)	48 (25.5%)		
Yes *n*(%)	259 (84.4%)	140 (74.5%)	0.51 (0.26–1.02)	0.057
PLT ≥ 300 × 10^9^L				
No *n*(%)	266 (86.6%)	77 (41%)		
Yes *n*(%)	41 (13.4%)	111 (59%)	8.45 (4.54–15.71)	< 0.001
HCY >7 umol/L				
No *n*(%)	259 (84.4%)	73 (38.8%)		
Yes *n*(%)	48 (15.6%)	115 (61.2%)	9.57 (5.18–17.70)	< 0.001

### Nomogram and prediction model evaluation

**Fig. 3:**
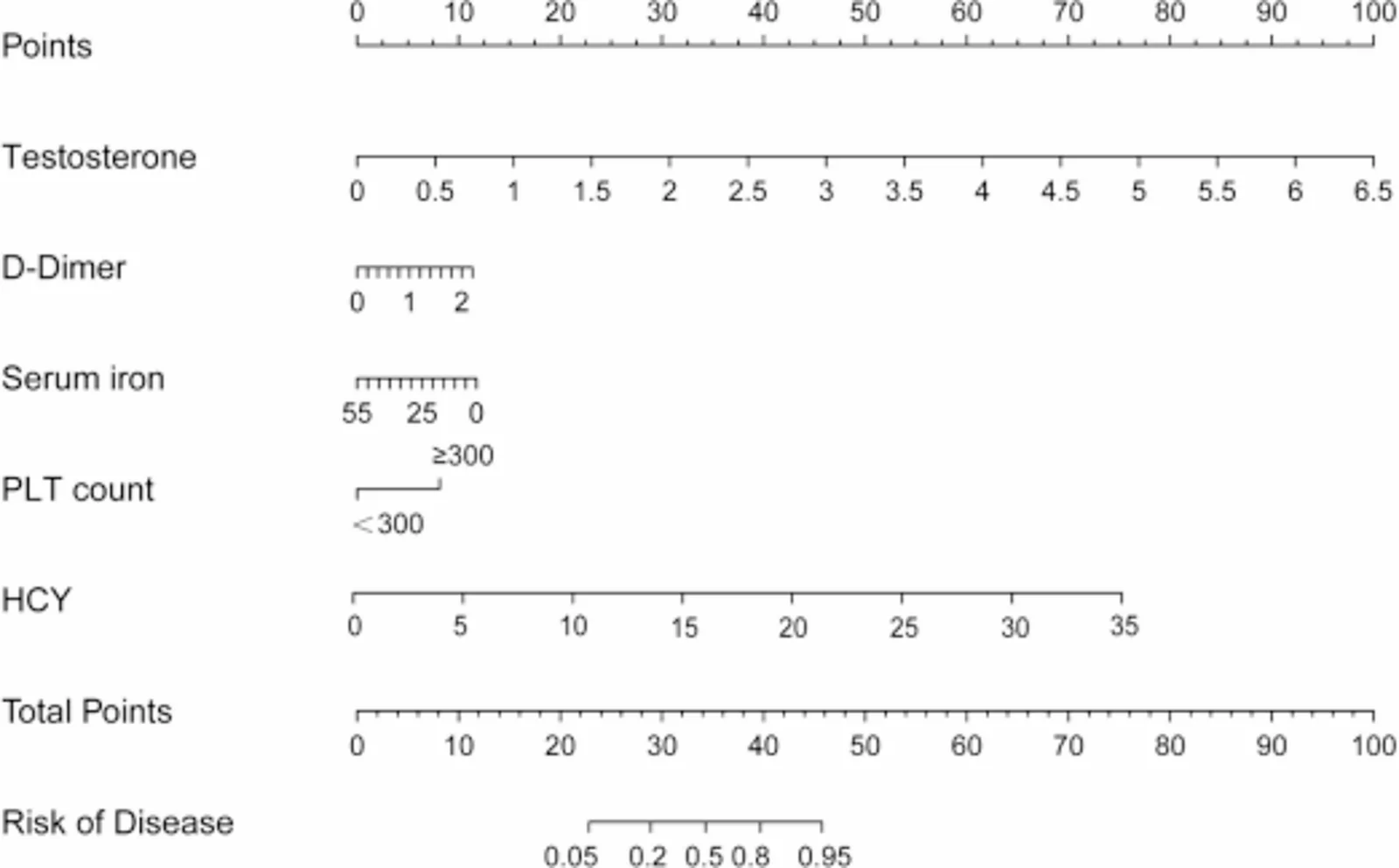
Nomogram for individualized estimation of early spontaneous abortion risk, showing point assignments for each predictor and the corresponding probability scale

A nomogram was developed incorporating five predictors: testosterone (0–6.5 nmol/L), D-dimer (0–4.5 mg/L), serum iron (0–2 µmol/L), PLT count (< 300 vs. ≥300 × 10⁹/L), and HCY (5–35 µmol/L). Each variable is assigned a score (0–100 points), and the total points correspond to a predicted probability of early spontaneous abortion (0.05–0.95) on the risk axis (Fig. 3).

**Fig. 4:**
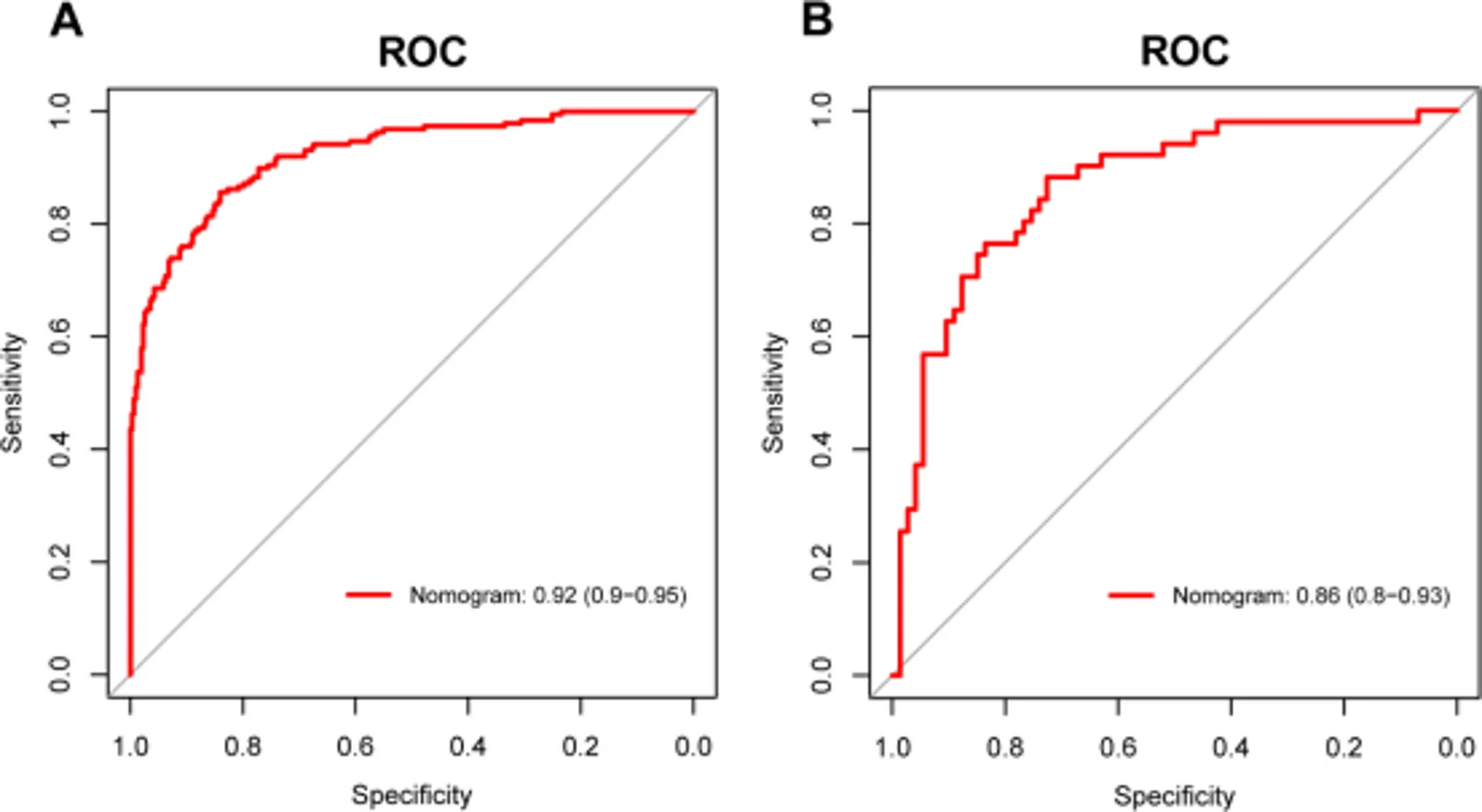
ROC curves illustrating the diagnostic trade-off between sensitivity and 1–specificity for the training (Fig. 4**A**) and validation (Fig. 4**B**) sets. A curve closer to the top-left corner indicates superior predictive performance

The nomogram demonstrated excellent discrimination in the training set, with an AUC of 0.92 (95% CI: 0.90–0.95) (Fig. 4A), indicating a strong ability to distinguish between patients with and without abortion. In the validation set, the model maintained good performance with an AUC of 0.86 (95% CI: 0.80–0.93) (Fig. 4B), supporting its generalizability.

**Fig. 5:**
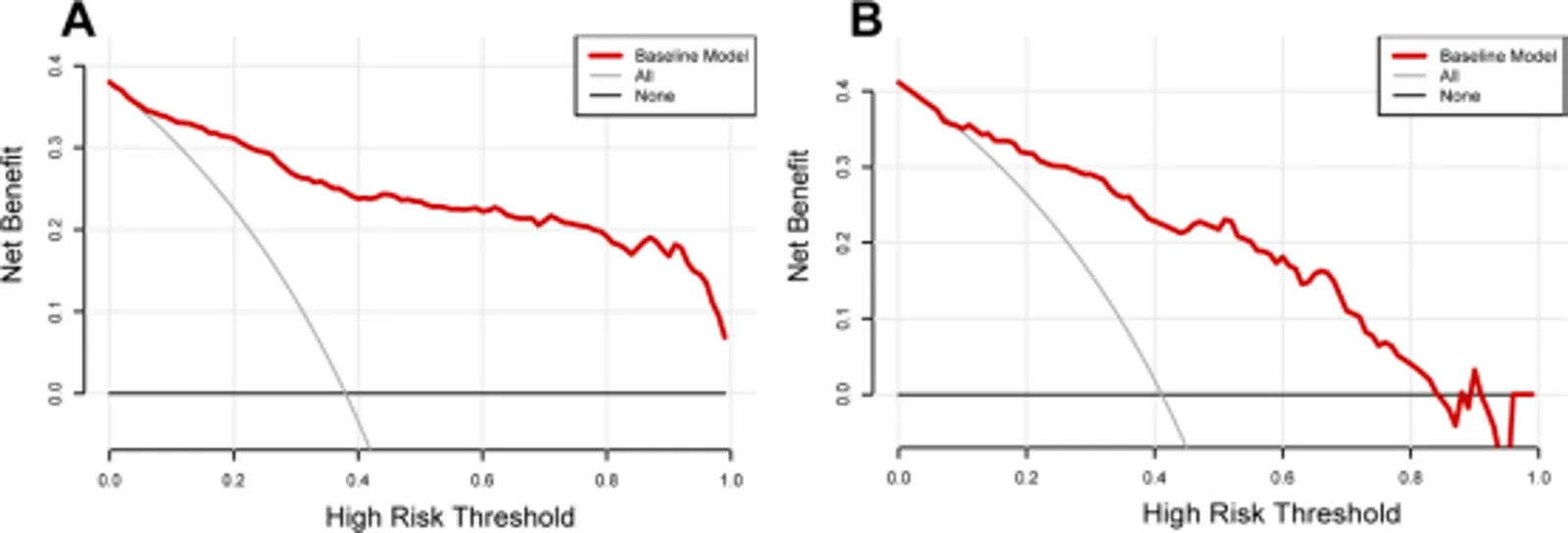
Decision Curve Analysis depicts the net benefit of the model across threshold probabilities for the training (Fig. 5**A**) and validation (Fig. 5**B**) sets. The net benefit of the model is compared against the strategies of intervening in all (“All”) or no (“None”) patients

Decision curve analysis (DCA) was employed to assess the model’s clinical utility. Across most threshold probabilities (0–1.0) in both the training (Fig. 5A) and validation (Fig. 5B) sets, the nomogram provided a superior net benefit compared to the “All” or “None” strategies, supporting its value for clinical decision-making.

**Fig. 6:**
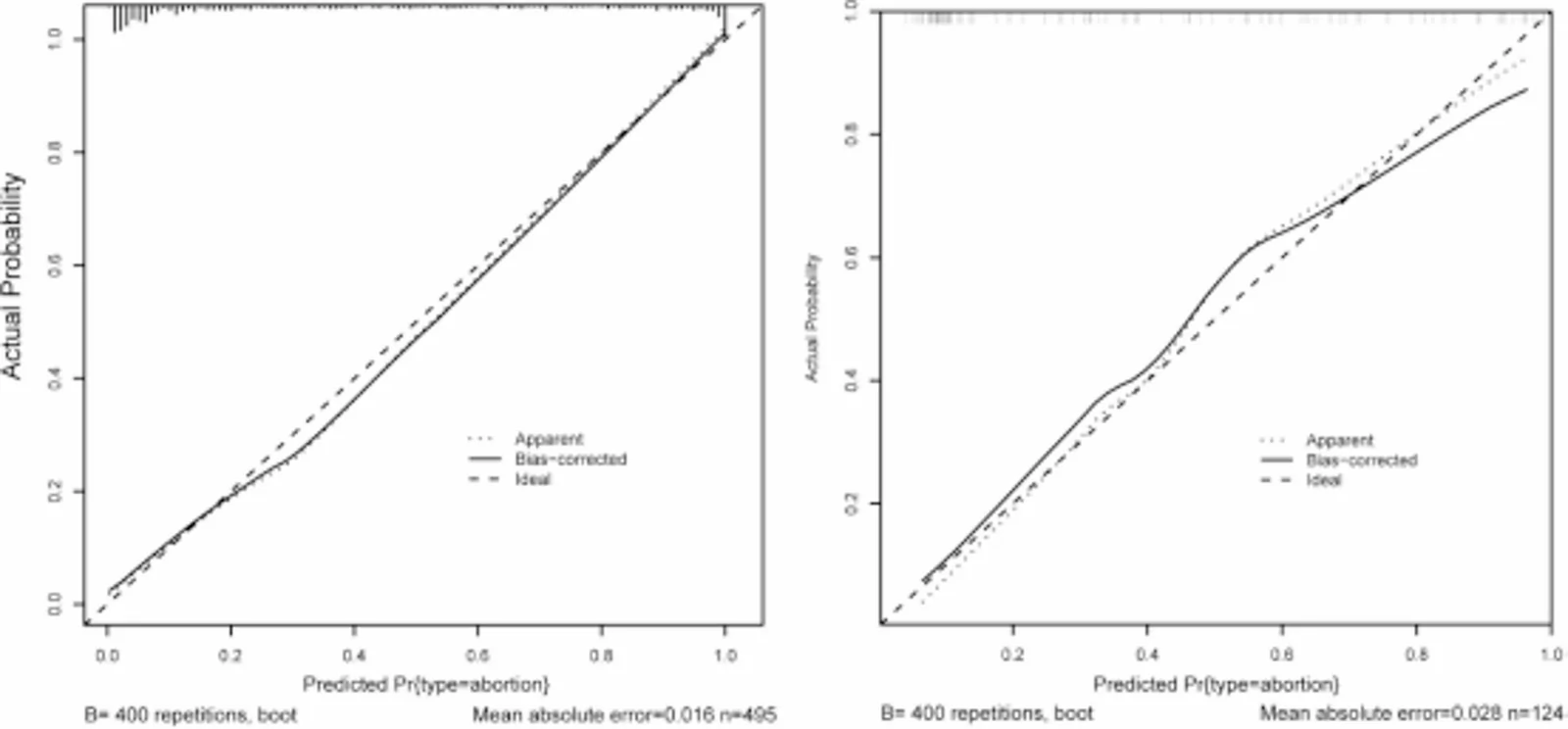
Calibration curves: Comparison of bootstrap-corrected (“Bias-corrected”) and ideal prediction lines for the training (Fig. 6**A**) and validation (Fig. 6**B**) sets, showing the relationship between predicted probabilities and actual event frequencies

The calibration performance was evaluated via bootstrap validation (400 resamples). In both the training (*n* = 495) and validation (*n* = 124) sets, the calibration curves closely aligned with the ideal line, with mean absolute errors of 0.016 and 0.028, respectively, demonstrating excellent agreement between predicted and observed probabilities (Fig. 6A, B).

## Discussion

This study utilized logistic regression analysis to identify independent risk factors for early spontaneous abortion in patients with PCOS and developed a predictive model accordingly. The model demonstrated excellent discriminative ability and calibration, showing strong potential for accurately identifying high-risk individuals. By facilitating the design of specialized and personalized prevention and intervention strategies, this tool may contribute to reducing the incidence of early spontaneous abortion in this vulnerable population.

PCOS is a complex disorder characterized by reproductive endocrine and metabolic dysfunction, often accompanied by a hypercoagulable state. Numerous factors associated with PCOS—including obesity, hyperandrogenism, insulin resistance, and a prothrombotic state—are closely linked to an increased risk of spontaneous abortion, both directly and indirectly affecting embryonic development through intricate interactions. Current research on risk factors for spontaneous abortion in PCOS has largely focused on reproductive hormones and metabolic aspects [8], with most studies examining only one or a limited number of variables [14]. There remains a lack of comprehensive and systematic analysis of the collective impact of multiple risk factors on abortion risk in this population. Our study addressed this gap by incorporating over ten potential predictors, including age, menstrual pattern, history of abortion, body mass index, thyroid function, LH/FSH ratio, testosterone, HOMA-IR, PLT count, HCY, coagulation profiles, lipid metabolism, serum iron levels, and early pregnancy support therapy. These variables encompass fundamental characteristics, endocrine profiles, glucolipid metabolism, coagulation function, and iron metabolism, allowing a multidimensional assessment of risk factors for spontaneous abortion in PCOS. Univariate analysis indicated that testosterone, HOMA-IR, D-dimer, serum iron, PLT count, HCY, and early pregnancy support therapy were significantly associated with early spontaneous abortion. Subsequent multivariate regression analysis revealed that elevated testosterone, D-dimer, PLT count, and HCY, along with decreased serum iron, remained independent risk factors for early pregnancy loss in PCOS after adjusting for confounding variables.

Hyperandrogenism, a core feature of PCOS, plays a critical role in the mechanisms underlying spontaneous abortion and adverse pregnancy outcomes. Patients with the hyperandrogenic phenotype of PCOS exhibit poorer endometrial receptivity and a significantly higher risk of miscarriage [17, 18]. Studies have identified 26 genes positively associated with recurrent implantation failure in hyperandrogenic PCOS patients, which are involved in corticosteroid metabolism, immune regulation, and bone metabolic processes [19]. Excess testosterone levels can downregulate the expression of insulin-like growth factor binding protein and leukemia inhibitory factor in the endometrium [20], inhibit the decidualization of endometrial stromal cells [21], and directly impair endometrial receptivity during early embryo implantation. Furthermore, hyperandrogenism significantly suppresses aromatase activity and induces mitochondrial dysfunction through androgen receptor-mediated pathways, leading to functional defects in endometrial cells [22–24]. Additionally, elevated testosterone and dehydroepiandrosterone increase the number of natural killer cells and activate inflammatory pathways and immune responses at the maternal-fetal interface. Such immune dysfunction may contribute to spontaneous abortion in PCOS [18, 19]. Hyperandrogenism and insulin resistance in PCOS can also trigger GPX4 deficiency-induced ferroptosis [25]. Ferroptosis not only drives fibrosis in endometrial epithelial cells, causing endometrial dysfunction, but also directly activates ERK/p38/JNK phosphorylation in the pregnant uterus and placenta, ultimately leading to abortion [26]. Hyperandrogenism is also an independent risk factor for late spontaneous abortion in PCOS, with the risk being 2.54 times higher in hyperandrogenic PCOS patients compared to those without hyperandrogenism [27]. This increased risk may be attributed to the co-occurrence of hyperandrogenism and cervical insufficiency. Preconception anti-androgen treatment has been shown to reduce the abortion rate from 18.92% to 9.46% before ovulation induction [28]. Consistent with these findings, our results demonstrated that elevated testosterone level was most strongly associated with abortion risk (OR = 50.11, 95% CI: 18.82–133.45, *P* < 0.001).

AMH, a key gonadotropin-independent regulator of follicular development and an indirect marker of the primordial follicle pool, has garnered increasing attention in PCOS research [29]. The 2023 international evidence-based guidelines recommend that AMH may be used as an alternative to antral follicle count in the diagnostic criteria for PCOS [2]. However, whether AMH is a risk factor for miscarriage in PCOS remains controversial. A study conducted in China demonstrated that among PCOS women aged 20–40, those with AMH levels between 4.98 and 10.65 ng/ml had a 1.592-fold higher risk of miscarriage compared to those with AMH ≤ 4.98 ng/ml, while those with AMH ≥ 10.65 ng/ml faced a 2.045-fold increased risk [30]. Notably, obesity—a modifiable factor—influences AMH levels [31], and elevated serum AMH was particularly associated with increased miscarriage risk in overweight/obese PCOS women [32]. In contrast, other studies have reported that preconception AMH levels do not affect perinatal outcomes in women with PCOS, even after adjustment for weight and age, suggesting no significant association with adverse perinatal events [32]. The risk of miscarriage related to AMH appears to be age-dependent, with younger PCOS patients exhibiting high AMH being more susceptible to early pregnancy loss, whereas no significant correlation has been observed in PCOS women over 35 years of age [33]. In line with the latter perspective, our study found that AMH > 7 ng/ml was not associated with an increased risk of early spontaneous abortion in PCOS patients.

In addition to its well-established effects on reproductive health, sex hormones play a central role in the pathogenesis of metabolic diseases. Hyperandrogenism promotes adipocyte dysfunction, visceral metabolic disorder, and exacerbated insulin resistance, and is considered a key metabolic driver in PCOS, engaging in a bidirectional and mutually reinforcing relationship with insulin resistance [34]. In PCOS, insulin resistance impairs endometrial receptivity by disrupting glucose transport in the endometrium and can induce mitochondrial dysfunction, leading to aberrant decidualization [23, 35]. Women with PCOS often exhibit significant dyslipidemia and sex hormone disturbances, with elevated BMI and androgen levels substantially increasing the risk of miscarriage [8]. A graded relationship has been observed between the HOMA-IR and abortion risk: early miscarriage rates were 7.14%, 13.21%, and 16.22% for HOMA-IR values ≤ 1.87, 1.87–4.28, and ≥ 4.28, respectively [36]. Central obesity, a characteristic feature of obesity in PCOS, significantly increases the likelihood of insulin resistance [37]. The waist-to-height ratio (WHtR) has been shown to be proportional to the degree of insulin resistance and serves as a reliable predictor of insulin resistance in PCOS [38]. Our results indicate that the prevalence of insulin resistance was significantly higher in PCOS patients who experienced spontaneous abortion compared to those who did not. Although BMI itself was not significantly associated with miscarriage in our cohort, we did not specifically examine the relationship between central obesity—a more precise reflection of metabolic risk—and insulin resistance, nor its direct effect on abortion. Consequently, after controlling for other variables in the multivariate analysis, insulin resistance did not emerge as an independent risk factor for miscarriage in PCOS.

Beyond contributing to endocrine and metabolic disturbances, PCOS is strongly associated with a high prevalence of coagulation disorders. Studies indicate that the rate of prethrombotic state among PCOS patients with recurrent spontaneous abortion reaches 70.7%, which is significantly higher than in non-PCOS patients [9]. A prethrombotic state is a well-established critical factor in recurrent pregnancy loss [39]. Our findings demonstrate that elevated levels of D-dimer, PLT count, and HCY significantly increase the risk of early spontaneous abortion in PCOS patients and remain independent risk factors even after adjusting for confounding variables. The prothrombotic state in PCOS arises from multiple interrelated mechanisms. First, PCOS patients exhibit hypercoagulability coupled with reduced fibrinolytic activity. Increased levels of plasminogen activator inhibitor-1 (PAI-1) inhibit the generation of plasmin, leading to impaired plasma fibrinolytic activity and a systemic hypercoagulable state [40]. Second, vascular endothelial dysfunction in PCOS promotes PLT activation and initiates coagulation pathways, directly contributing to a prethrombotic state [41]. PLT also play a pivotal role in the thrombophilic tendency associated with PCOS. Patients show enhanced PLT aggregation and increased PLT count, which not only facilitate thrombus formation but also release multiple bioactive substances, such as platelet-derived microparticles [42]. These microparticles further activate the coagulation system and have been positively correlated with serum testosterone levels [43]. Moreover, the platelet-to-lymphocyte ratio (PLR), a novel inflammation-derived biomarker reflective of infection, inflammatory response, or immune status, is significantly elevated during thrombotic events [44, 45]. PCOS patients with adverse pregnancy outcomes are at a higher risk of increased PLR [46]. Similarly, the systemic immune-inflammation index (SII) integrates pathways related to thrombosis, inflammation, and immune response, serving as a comprehensive indicator of overall immune and inflammatory status [47]. These markers highlight the interplay between inflammatory response and PLT activation, further promoting the development of a prethrombotic state in PCOS and consequently increasing the risk of miscarriage [47].

Hyperhomocysteinemia is an independent risk factor for recurrent spontaneous abortion. Patients with PCOS exhibit significantly higher HCY levels than non-PCOS individuals, and these levels are positively correlated with body mass index, free testosterone, and fasting blood glucose [48]. Elevated HCY markedly increases the risk of miscarriage in PCOS [49]. There is evidence of an interaction between hyperhomocysteinemia and hyperandrogenism, which collectively disrupt lipid homeostasis and consequently affect coagulation function in PCOS [48]. Moreover, hyperhomocysteinemia is also an independent risk factor for metabolic syndrome [50]. It may promote insulin resistance and adipose tissue inflammation in PCOS by modulating macrophage M1/M2 polarization via suppression of estrogen signaling [51]. These metabolic disturbances, including insulin resistance, can further promote PLT activation and lead to coagulation abnormalities [52]. Notably, although insulin sensitizers can improve insulin resistance in PCOS patients, they may also raise HCY levels, highlighting the need for careful risk-benefit assessment and individualized treatment strategies in clinical practice [53].

Iron is an essential nutrient for maintaining pregnancy, playing a critical role in placental development, fetal oxygen supply, and immune regulation [54]. During pregnancy, maternal iron demand increases significantly, and inadequate intake or absorption may lead to iron deficiency, thereby increasing the risk of adverse pregnancy outcomes through multiple mechanisms [54, 55]. Consistent with previous reports [56], our results indicate that serum iron levels are significantly lower in patients with recurrent pregnancy loss compared to those with normal early pregnancies, and we identified reduced serum iron as an independent risk factor for early spontaneous abortion in PCOS. Interestingly, earlier studies reported that ferritin levels are significantly higher in women with PCOS than in non-PCOS women (51.43 µg/L vs. 44.85 µg/L) [57]. Reduced menstrual blood loss due to oligo- or amenorrhea and elevated androgen levels are proposed contributors to increased ferritin in PCOS [57]. As ferritin is a key iron storage protein, elevated ferritin may lead to increased cellular iron retention while limiting iron export, resulting in lower circulating serum iron levels. However, the precise iron metabolism pathways involved in this process require further elucidation.

Furthermore, some studies have suggested that compared to PCOS patients with regular menstruation, amenorrhea is a risk factor for adverse pregnancy outcomes and miscarriage. However, after adjusting for confounding factors, amenorrhea remained an independent risk factor for adverse pregnancy outcomes but not for miscarriage, which is consistent with our findings that menstrual pattern may not significantly influence the risk of abortion in PCOS [58]. Our study also indicates that early pregnancy support therapy may reduce the risk of spontaneous abortion, although this result did not reach statistical significance. It should be noted that our research did not detail the specific types or medications used for pregnancy support. Therefore, future studies should focus on evaluating the effects of different pregnancy support strategies on abortion risk in women with PCOS.

## Conclusion

This study identified elevated testosterone, D-dimer, PLT count, and HCY, along with decreased serum iron, as independent risk factors for early spontaneous abortion in women with PCOS. A nomogram incorporating these factors was developed to predict the risk of early pregnancy loss and demonstrated high predictive accuracy. This tool shows strong potential to support individualized clinical decision-making and facilitate early intervention strategies for high-risk PCOS patients.

### Limitations

A limitation of our prediction model is that it was developed using single-center, retrospective data. Although it demonstrated good performance through internal validation, including train-test split and bootstrap resampling, its generalizability necessitates further validation in future prospective, multi-center, large-scale cohorts.

## Supplementary Information

Supplementary Material 1.

Supplementary Material 2.

## Data Availability

The raw data supporting the conclusions of this article will be made available by the corresponding author upon reasonable request.

## Abbreviations

PCOS: Polycystic ovary syndrome
BMI: Body mass index
AMH: Anti-Müllerian hormone
EP: Ectopic pregnancy
GTD: Gestational trophoblastic disease
HIS: Hospital Information System
LH/FSH: Luteinizing hormone/follicle-stimulating hormone
HOMA-IR: Homeostasis model assessment of insulin resistance
TSH: Thyroid-stimulating hormone
FIB: Fibrinogen
PT: Prothrombin time
PA: Prealbumin
TT: Thrombin time
APTT: Activated partial thromboplastin time
PLT: Platelet
HCY: Homocysteine
LASSO: Least absolute shrinkage and selection operator
ROC: Receiver operating characteristic
AUC: Area under the curve
Coef: Coefficient
DCA: Decision curve analysis
WHtR: Waist-to-height ratio
PAI-1: Plasminogen activator inhibitor-1
PLR: Platelet-to-lymphocyte ratio
SII: Immune-inflammation index
