# Supplementary material for: A nomogram to predict early spontaneous abortion in women with polycystic ovary syndrome: a retrospective cohort study
Source: BMC Pregnancy Childbirth. 2026 Jul 10;26:1108. doi: 10.1186/s12884-026-09641-w (PMC13637341; doi:10.1186/s12884-026-09641-w)
Supplement: Supplementary file 1 [file 12884_2026_9641_MOESM1_ESM.pdf]

首都医科大学附属北京妇产医院医学伦理委员会

伦理审查批件

审查决定文件编号/伦理审查批件号	2025-KY-047-01		
项目名称	多囊卵巢综合征早期自然流产风险预测模型		
申办单位	首都医科大学附属北京妇产医院		
本院申请部门	中医科	项目负责人	韩倩

伦理委员会声明：

* 本伦理委员会按照我国涉及人的生物医学研究、医疗保健技术等伦理审查相关法规、政策与指南及相关国际伦理准则组成和工作，
* 本伦理委员会的组成和工作相对独立，
* 本伦理委员会对所审阅的项目资料和相关内容遵守保密制度和研究利益冲突政策，

审查方式	快速审查
审查类别	初始审查
审查时间	2025-05-06

审查文件清单：（含版本号、版本日期）

1.初始审查申请；2.研究方案（版本号: 20250410; 版本日期: 20250410);

3.知情同意书（版本号: 20250410; 版本日期: 20250410);

4.研究者遵照相关要求开展病历回顾性研究项目的声明；

5.科技处课题申请书；6.科技处立项证明；7.研究材料诚信承诺书

1. 伦理委员会审查决定：批准。
2. 该项目进行过程中将接受伦理委员会的定期跟踪审查？【√】是；【】否

   定期跟踪审查频率： 12 个月
3. 请于【 2026 年 04 月 06 日之前】递交年度/定期跟踪审查，
4. 本批件自审签之日起生效，有效期与跟踪审查频率相同，

主任委员或其授权人签字：

首都医科大学附属北京妇产医院医学伦理委员会（盖章）：

日期： 2025 年 05 月 06 日

说明：

1. 请按照我国涉及人的生物医学研究、医疗保健技术等相关法规、政策与指南及相关国际伦理准则、伦理委员会批准的文件开展研究，保护受试者的健康与权益。
2. 凡涉及人类遗传资源管理或者国家规定必须经有关部门审批的项目，均需在执行前向有关部门申报并获得批准。
3. 项目开展过程中，如对已批准的项目资料做任何修改，请按照修正案审查申请提交相关资料，获得批准后方可执行。
4. 项目发生安全性事件或影响受试者/患者安全或权益的事件，请按国家规定向监管部门、伦理委员会等相关部门报告。
5. 项目负责人应按照伦理委员会规定的跟踪审查频率，在截止日期前1个月（无论研究开始与否）提交年度/定期跟踪审查申请。
6. 本中心发生违背方案时，请及时提交违背方案审查申请。
7. 暂停或提前终止的项目，请及时提交暂停或终止研究审查申请。
8. 项目完成或结题，请提交研究完成或总结审查申请。
9. 请在批件有效期内开展项目，逾期未开展的，本批件失效。
10. 伦理批件失效后的项目，再次开展时，需重新伦理审查。
11. 如对伦理委员会的审查结果有疑问或需要申诉，请联络医院伦理委员会，并提交书面申诉意见，详细说明申诉理由。
12. 本伦理委员会审查申请相关信息，详见本院官网-伦理委员会网站信息。
13. 如为会议审查，本文件附会议审查委员签到表。
14. 伦理委员会办公室地址：北京市朝阳区团结湖北五条团结湖街道社区服务中心201室（100026）。
15. 伦理委员会联系方式：办公室电话/传真：010-85968407；Email：fcyylunli@163.com。
