# Supplementary material for: A nomogram to predict early spontaneous abortion in women with polycystic ovary syndrome: a retrospective cohort study
Source: BMC Pregnancy Childbirth. 2026 Jul 10;26:1108. doi: 10.1186/s12884-026-09641-w (PMC13637341; doi:10.1186/s12884-026-09641-w)
Supplement: Supplementary file 2 [file 12884_2026_9641_MOESM2_ESM.doc]

****A Nomogram to Predict Early Spontaneous Abortion in Women with Polycystic Ovary Syndrome: A Retrospective Cohort Study****

Qian Han^1,2^, Mengyuan Li^1^, Guanshan Li^1,2^, Mingwei Xin^1^, Xiaodan Yin^1^, Junqin He^1^

^1^Department of Traditional Chinese Medicine, Beijing Obstetrics and Gynecology Hospital, Capital Medical University. Beijing Maternal and Child Health Care Hospital，Beijing，China

^2^Capital Medical University，Beijing，China

Qian Han, No. 251 Yaojiayuan Road, Chaoyang District, Beijing,China(100026),

Email:[hanqian@mail.ccmu.edu.cn](mailto:hanqian@mail.ccmu.edu.cn); ORCID iD: 0009-0005-5847-5388

Mengyuan Li, No. 251 Yaojiayuan Road, Chaoyang District, Beijing,China(100026),

Email:limengyuan@ccmu.edu.cn

Guanshan Li, No. 251 Yaojiayuan Road, Chaoyang District, Beijing,China(100026),

Email:296186953@qq.com

Mingwei Xin, No. 251 Yaojiayuan Road, Chaoyang District, Beijing,China(100026),

Email:xinmingwei@ccmu.edu.cn

Xiaodan Yin, No. 251 Yaojiayuan Road, Chaoyang District, Beijing,China(100026),

Email:yinxiaodandong@ccmu.edu.cn

Junqin He, No. 251 Yaojiayuan Road, Chaoyang District, Beijing,China(100026),

Email:hejunqin@ccmu.edu.cn；ORCID iD: 0000-0002-3223-7710

**Corresponding author**

Prof. Junqin He, PhD,Department of Beijing Obstetrics and Gynecology Hospital,Capital Medical University, No. 251 Yaojiayuan Road, Chaoyang District, Beijing,100026, Email:hejunqin@ccmu.edu.cn.

**Word counts :** 5564

**The number of tables:** 3

**The number of figures:** 6

**Grant support or other sources of funding:** This study was supported by the Beijing Capital Development Project of Science and Technology(Grant No:SHF2024-2-2114) and the Natural Science Foundation of Beijing (Grant No: 7234408).

**Declarations**

**Ethics approval and consent to participate:** This study was conducted in strict accordance with the ethical guidelines of the Declaration of Helsinki. Ethical approval was obtained from the Ethics Committee of Beijing Obstetrics and Gynecology Hospital, Capital Medical University (Approval Number: 2025-KY-047-01). This study involved a retrospective analysis of clinical data using de-identified data, and did not involve personal privacy or commercial interests. Therefore, the Ethics Committee waived the requirement for written informed consent.

**Consent for publication:** Not applicable.

**Competing interests:** The authors declare that they have no competing interests.
